# Guideline-informed vancomycin therapeutic drug monitoring indications in an adult intensive care unit: a retrospective descriptive study

**DOI:** 10.1186/s12871-026-04078-2

**Published:** 2026-07-06

**Authors:** Yunus Emre Ayhan, Rabia Sarı Küçük, Aslı Nur Özkan, Hilal Akay Gedi̇k, Funda Şi̇mşek, Muhammed Yunus Bektay

**Affiliations:** 1 Prof. Dr. Cemil Taşcıoğlu City Hospital, Clinical Pharmacy, Istanbul, Türkiye; 2 Department of Intensive Care, Prof. Dr. Cemil Taşcıoğlu City Hospital, Istanbul, Türkiye; 3 Department of Anesthesiology and Reanimation, Prof. Dr. Cemil Taşcıoğlu City Hospital, Istanbul, Türkiye; 4 Department of Infectious Disease and Clinical Microbiology, Prof. Dr. Cemil Taşcıoğlu City Hospital, Istanbul, Türkiye; 5 Department of Clinical Pharmacy, Faculty of Pharmacy, Istanbul University-Cerrahpasa, Istanbul, Türkiye

**Keywords:** Vancomycin, Therapeutic drug monitoring, Intensive care unit, Pharmacokinetics, Antimicrobial stewardship

## Abstract

**Background:**

Vancomycin is widely used in intensive care units (ICUs) for severe Gram-positive infections, particularly those caused by methicillin-resistant *Staphylococcus aureus* (MRSA). Because critically ill patients have high pharmacokinetic variability and toxicity risk, therapeutic drug monitoring (TDM) is recommended in selected high-risk conditions. However, real-world data quantifying guideline-informed TDM eligibility in ICUs where TDM is not available remain limited. This study aimed to estimate the burden and criterion-level profile of TDM eligibility among adult ICU patients receiving vancomycin.

**Methods:**

A retrospective descriptive study was conducted in an adult ICU between January 1, 2024, and July 1, 2025. TDM eligibility was assessed using guideline-derived domains from the 2020 IDSA/ASHP/PIDS/SIDP consensus recommendations, operationalized for retrospective ICU assessment. Because vancomycin TDM was not performed during the study period, drug concentrations, area under the curve (AUC) estimates, target attainment, and TDM-guided dose modifications were unavailable. Data were summarized descriptively, and between-group comparisons were performed using chi-square test, Fisher’s exact test, and Mann-Whitney U test, as appropriate.

**Results:**

During the study period, 341 of 1894 ICU admissions (18.0%) involved vancomycin therapy, and 331 patients were included in the final analysis. Overall, 319 patients (96.4%) met at least one TDM indication criterion. The most frequent indications were prolonged therapy, defined as observed treatment duration exceeding 5 days during the treatment course (276, 83.4%), altered pharmacokinetics (227, 68.6%), renal dysfunction (180, 54.4%), nephrotoxic co-administration (174, 52.6%), and serious or invasive MRSA infection (25, 7.6%). Indications frequently overlapped, with a median of 3 (IQR: 2–4) criteria per patient.

**Conclusion:**

Nearly all vancomycin-treated ICU patients met guideline-informed TDM eligibility criteria, indicating a substantial burden of monitoring eligibility in this Turkish ICU where TDM was not available. These findings primarily inform local resource planning, but may also be relevant to institutions with similar reimbursement, laboratory, or infrastructure constraints. Prospective pre-post implementation studies should evaluate target attainment, nephrotoxicity, clinical outcomes, and costs.

**Supplementary Information:**

The online version contains supplementary material available at https://doi.org/10.1186/s12871-026-04078-2.

## Background

Methicillin-resistant *Staphylococcus aureus* (MRSA) remains a major cause of severe infections, particularly among critically ill patients admitted to intensive care units (ICUs), where vancomycin continues to serve as a cornerstone of therapy [1, 2]. Despite its widespread clinical use, vancomycin has a narrow therapeutic index, and both subtherapeutic and supratherapeutic exposures are associated with adverse outcomes, including treatment failure, the emergence of antimicrobial resistance, and nephrotoxicity [3–5]. Critically ill patients are particularly prone to pharmacokinetic variability due to dynamic physiological alterations such as augmented renal clearance, fluid shifts, hypoalbuminemia, and the use of extracorporeal therapies, all of which contribute to unpredictable drug exposure [6–8]. As a result, therapeutic drug monitoring (TDM) has been widely recommended to optimize vancomycin dosing, improve clinical outcomes, and minimize toxicity [9–11].

The 2020 consensus guideline jointly published by the Infectious Diseases Society of America (IDSA), American Society of Health-System Pharmacists (ASHP), Pediatric Infectious Diseases Society (PIDS), and Society of Infectious Diseases Pharmacists (SIDP) introduced a major shift in vancomycin monitoring, recommending area under the concentration-time curve (AUC)-guided dosing instead of trough-based monitoring, with a target AUC to minimum inhibitory concentration (MIC) ratio of 400–600 mg*h/L [12–14]. This recommendation is supported by growing evidence demonstrating that AUC-guided strategies are superior in predicting both efficacy and nephrotoxicity compared with trough-based approaches [15–17]. Despite these recommendations, the implementation of vancomycin TDM in routine clinical practice remains inconsistent across healthcare settings. Barriers such as limited access to Bayesian software, inadequate laboratory infrastructure, and lack of trained clinical pharmacists restrict the use of AUC-guided monitoring, particularly in resource-limited environments [14, 16, 18]. Consequently, empirical dosing strategies without systematic monitoring are still commonly used. In this institutional practice, vancomycin drug-level assays were not reimbursed or covered during the study period, and no dedicated institutional funding was available for routine testing. As a result, neither trough-based nor AUC-guided monitoring was systematically applied, and vancomycin dosing relied on empirical strategies without individualized pharmacokinetic adjustment.

Although guideline-informed indications for vancomycin TDM are well established, real-world data quantifying the burden of TDM eligibility in ICU settings remain limited. This issue is particularly important in institutions where vancomycin TDM is not performed and resources are constrained. Therefore, the present study aimed to determine the proportion of patients meeting guideline-informed criteria for vancomycin TDM eligibility in an adult ICU without vancomycin TDM. This study evaluates eligibility for TDM, not actual TDM performance, AUC target attainment, dose adjustment, nephrotoxicity, microbiological response, treatment failure, mortality, or economic outcomes attributable to TDM. In addition, the study aimed to describe exploratory clinical correlates of TDM indication status, thereby providing real-world evidence to support resource planning and implementation strategies in intensive care settings. This study provides real-world data from Türkiye on guideline-informed vancomycin TDM eligibility in an adult ICU setting where vancomycin TDM is not performed, contributing to the limited evidence base from resource-constrained settings.

## Methods

### Study design and participants

This study was designed as a retrospective, descriptive study conducted in the anesthesia and reanimation ICU of a tertiary care hospital. Patients admitted between January 1, 2024, and July 1, 2025, were screened for eligibility.

Patients aged ≥ 18 years who were hospitalized in the ICU for at least 24 h and received vancomycin for any clinical indication were included in the study. All eligible patients were evaluated in terms of vancomycin TDM indications. Patients with incomplete or missing data were excluded from the analysis.

### Data collection

Data were retrospectively obtained from the hospital’s electronic medical records system. Collected variables included demographic characteristics, length of ICU stay, comorbidities, and clinical history, as well as detailed information on concomitant medications. Laboratory parameters, including serum creatinine levels and calculated creatinine clearance, were recorded to assess renal function. Information regarding renal replacement therapy, mechanical ventilation (MV) status, and microbiological culture results was also extracted. In addition, acute phase reactants, indications for vancomycin therapy, dosage, and duration of treatment were collected. All relevant clinical and laboratory data were reviewed to ensure a comprehensive evaluation of patients receiving vancomycin during their ICU stay.

### Assessment of vancomycin TDM indications

TDM eligibility criteria were operationalized for retrospective ICU assessment using guideline-derived domains from the 2020 consensus recommendations published by the Infectious Diseases Society of America in collaboration with ASHP, PIDS, and SIDP. The consensus recommendations primarily focus on serious MRSA infections but also identify patient groups in whom monitoring is warranted because of renal dysfunction, nephrotoxicity risk, prolonged therapy, or critical illness-related pharmacokinetic variability; therefore, these domains were translated into routinely documented ICU variables [12]. During the study period, vancomycin TDM was not performed in this institution. Therefore, no included patient underwent vancomycin TDM, and actual vancomycin concentrations, AUC estimates, target attainment, and TDM-guided dose modifications were not available for analysis. Patients were evaluated according to predefined clinical criteria reflecting established indications for vancomycin TDM. These criteria included serious or invasive MRSA infection (I1), impaired or unstable renal function such as acute kidney injury or the requirement for renal replacement therapy (I2), and concomitant use of nephrotoxic agents as defined by the guideline, specifically aminoglycosides, amphotericin B, or intravenous contrast agents (I3). Other agents with nephrotoxic potential but not included in this operational definition were not counted under I3. Additional criteria included prolonged vancomycin therapy, defined as observed treatment duration exceeding 5 days during the treatment course (I4), and clinical conditions associated with altered pharmacokinetics such as sepsis, burn injury, or hemodynamic instability (I5). Because of the retrospective design, acute kidney injury was identified based on routine physician documentation in the medical records and available renal function parameters, including serum creatinine trends and the need for renal replacement therapy. CRRT was classified under I2 and was not considered an additional I5 criterion solely by itself. For I5, sepsis and hemodynamic instability were determined retrospectively according to physicians’ routine clinical assessments documented in the medical records and based on standard guideline-informed clinical definitions. Hemodynamic instability was considered present when the treating physicians documented hemodynamic instability or when vasopressor support was required. Augmented renal clearance, obesity, ECMO, and hypoalbuminemia were not formally assessed because of data availability constraints. Given the retrospective design, I4 was assessed during the treatment course based solely on observed treatment duration documented in medical records and was not interpreted as an indication identifiable at vancomycin initiation. Empirical versus targeted therapy classification was assigned retrospectively based on whether microbiological culture results were available and guided the vancomycin prescription at the time of initiation or continuation.

Patients meeting at least one of these criteria were considered eligible for TDM. Since multiple indications could coexist in a single patient, each criterion was recorded individually and analyzed both separately and in combination.

### Definitions

Vancomycin therapy was categorized as either empirical or targeted (culture-guided). Empirical therapy was defined as vancomycin initiated for suspected infection in the absence of microbiological confirmation at the time of treatment initiation. Targeted therapy was defined as vancomycin treatment initiated or continued based on microbiologically confirmed infection and culture susceptibility results.

### Sample size

The required sample size was calculated based on the total population of 341 patients who received vancomycin during the study period. Assuming a 95% confidence level, a 5% margin of error, and a 50% expected proportion (representing the most conservative estimate), the minimum required sample size was calculated as 181 patients. However, to better reflect real-world clinical practice and to increase the precision of the estimates, all eligible patients were included in the final analysis.

### Statistical analysis

Statistical analyses were performed using IBM SPSS Statistics for Windows, Version 25.0 (IBM Corp., Armonk, NY, USA). Categorical variables were compared using the Chi-square test or Fisher’s exact test, as appropriate. Continuous variables were assessed for normality using the Shapiro–Wilk test. As the data were not normally distributed, comparisons between groups were performed using the Mann–Whitney U test. Descriptive statistics were presented as median (interquartile range [IQR]) for continuous variables and as frequency and percentage [n (%)] for categorical variables.

Given the extreme imbalance between groups (TDM indicated: *n* = 319, 96.4%; TDM not indicated: *n* = 12, 3.6%) and the near-universal presence of TDM indications, multivariable logistic regression modelling was not performed, as it would lack adequate statistical power and discriminatory capacity. Between-group comparisons were therefore limited to descriptive and bivariate analyses, and a p value < 0.05 was considered statistically significant.

## Results

**Fig. 1:**
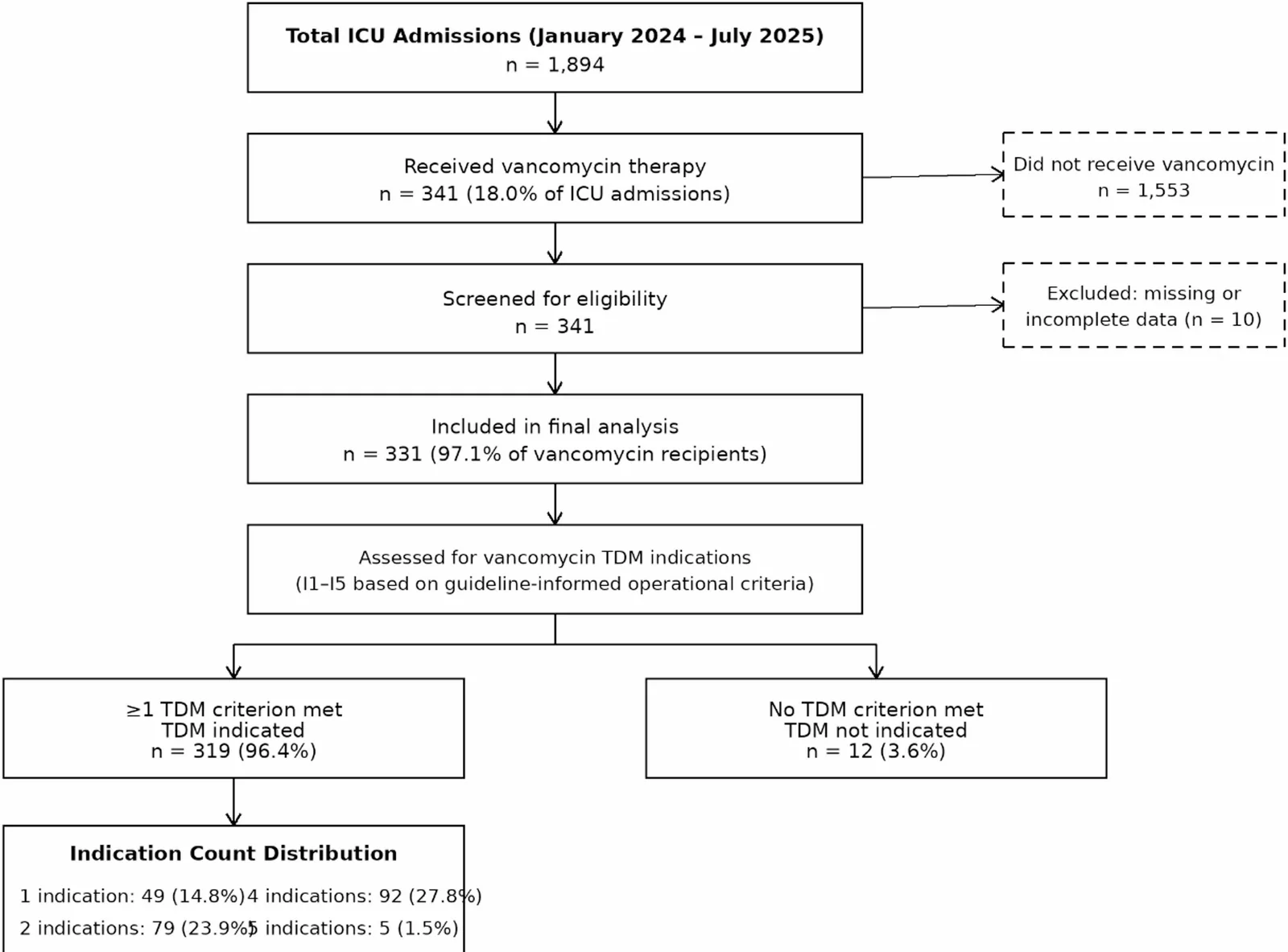
Study flowchart

A total of 1894 patients were admitted to the anesthesia ICU between January 1, 2024, and July 1, 2025. Among these, 341 patients (18.0%) received vancomycin therapy. After excluding 10 patients due to missing data, 331 patients (97.1%) with complete data were included in the final analysis (Fig. 1).

Baseline sociodemographic and clinical characteristics of the study population are presented in Table 1. The median age of the patients was 67.0 years (IQR: 54.0–76.0), and 58.9% were male. The majority of admissions were for medical reasons (75.2%). The median ICU length of stay was 14.0 days (IQR: 6.0–28.0), and most patients required MV (81.6%). Common comorbidities included hypertension (44.7%), malignancy (29.3%), diabetes mellitus (27.8%), and coronary artery disease (25.1%). The median number of comorbidities was 2.0 (IQR: 1.0–3.0). The most frequent reasons for ICU admission were respiratory failure (25.1%) and postoperative care (24.8%).

**Table 1:** Sociodemographic and clinical characteristics of ICU patients

Variable	Total (*n* = 331)	TDM Indicated (*n* = 319)	TDM Not Indicated (*n* = 12)	*p* value
Demographics				
Sex, n (%)				1.000
Male	195 (58.9)	188 (58.9)	7 (58.3)	
Female	136 (41.1)	131 (41.1)	5 (41.7)	
Age (years), median (IQR)	67.0 (54.0–76.0)	68.0 (54.0–76.0)	59.5 (47.8–71.5)	0.195
Admission Characteristics				
ICU length of stay (days), median (IQR)	14.0 (6.0–28.0)	14.0 (6.0–28.5)	7.0 (2.0–9.0)	**0.007**
Admission type, n (%)				0.501
Medical	249 (75.2)	241 (75.5)	8 (66.7)	
Surgical	82 (24.8)	78 (24.5)	4 (33.3)	
Comorbidities, n (%)				
Hypertension	148 (44.7)	—	—	—
Malignancy	97 (29.3)			
Diabetes mellitus	92 (27.8)			
Coronary artery disease	83 (25.1)			
Chronic kidney disease	42 (12.7)			
Chronic obstructive pulmonary disease	40 (12.1)			
Cerebrovascular disease	30 (9.1)			
Dementia / Alzheimer’s disease	27 (8.2)			
Atrial fibrillation / arrhythmia	22 (6.6)			
Other	88 (26.6)			
Reason for ICU Admission, n (%)ᵇ		—	—	—
Respiratory failure/distress	83 (25.1)	—	—	—
Postoperative / post-surgical	82 (24.8)			
Decreased consciousness	25 (7.4)			
Inotropic support requirement	23 (6.8)			
Sepsis / septicemia	23 (6.8)			
Pneumonia / pneumosepsis	19 (5.6)			
Trauma / multiple fractures	16 (4.7)			
Subarachnoid hemorrhage	12 (3.6)			
Intracranial hemorrhage	8 (2.4)			
Hypotension	7 (2.1)			
Other	39 (11.6)			
Number of comorbidities, median (IQR)	2.0 (1.0–3.0)	2.0 (1.0–3.0)	2.0 (1.0–3.0)	0.827
Clinical Parameters				
Mechanical ventilation, n (%)	270 (81.6)	265 (83.1)	5 (41.7)	**0.001**
MV duration (days), median (IQR)	10.0 (3.0–24.0)	11.0 (3.0–24.0)	0.5 (0.0–3.0)	**0.003**
Sepsis/septic shock, n (%)	250 (75.5)	247 (77.4)	3 (25.0)	**< 0.001**
Central venous catheter, n (%)	263 (79.5)	260 (81.5)	3 (25.0)	**< 0.001**
Outcomes				
Mortality, n (%)	226 (68.3)	221 (69.3)	5 (41.7)	0.089
CRRT, n (%)	111 (33.5)	111 (34.8)	0 (0.0)	**0.010**

Percentages for comorbidities were calculated using the total cohort as the denominator (*n* = 331). Because patients could have more than one comorbidity or more than one reason for ICU admission, category totals may exceed 100%

Categorical variables were compared using Chi-square or Fisher’s exact test; continuous variables using Mann-Whitney U test

*IQR* Interquartile range, *MV *Mechanical ventilation, *CRRT *Continuous renal replacement therapy, *TDM *Therapeutic drug monitoring

Bold *p* values indicate statistical significance (*p* < 0.05)

Microbiological and vancomycin treatment characteristics are summarized in Table 2. Culture positivity was observed in 44.4% of patients, while 55.6% had negative cultures. Among culture-positive patients (*n* = 147), the most commonly isolated pathogens were coagulase-negative staphylococci (MRCNS; 29.9%), *Enterococcus faecium* (21.8%), MRSA (17.0%), and *Enterococcus faecalis* (12.2%). Vancomycin was used empirically in 59.5% of patients and as targeted (culture-guided) therapy in 40.5%. The median duration of vancomycin therapy was 6.0 days (IQR: 4.0–12.0), and the median daily dose was 2.0 g (IQR: 1.0–2.0 g).

**Table 2:** Culture, microbiology, and vancomycin treatment characteristics

Variable	Total (*n* = 331)	TDM Indicated (*n* = 319)	TDM Not Indicated (*n* = 12)	*p* value
Culture and Microbiology				
Culture obtained, n (%)	315 (95.2)	303 (95.0)	12 (100.0)	1.000
Culture not obtained, n (%)	16 (4.8)	16 (5.0)	0 (0.0)	1.000
Positive culture, n (%)	147 (44.4)	147 (46.1)	0 (0.0)	**0.002**
Negative culture, n (%)	184 (55.6)	172 (53.9)	12 (100.0)	**0.002**
Most Common Isolated Pathogens (among culture-positive patients, *n* = 147)				
MRCNS, n (%)	44 (29.9)	—	—	—
Enterococcus faecium, n (%)	32 (21.8)	—	—	—
MRSA isolates, n (%)ᵃ	25 (17.0)	—	—	—
Enterococcus faecalis, n (%)	18 (12.2)	—	—	—
Vancomycin Treatment				
Empirical treatment, n (%)	197 (59.5)	185 (58.0)	12 (100.0)	**0.002**
Targeted treatment, n (%)	134 (40.5)	134 (42.0)	0 (0.0)	**0.002**
Vancomycin duration (days), median (IQR)	6.0 (4.0–12.0)	6.0 (4.0–12.0)	2.0 (1.0–2.0)	**< 0.001**
Vancomycin daily dose (g), median (IQR)	2.0 (1.0–2.0) g	2.0 (1.0–2.0) g	2.0 (2.0–2.0) g	0.104
Additional Clinical Features				
Concomitant nephrotoxin use, n (%)	179 (54.1)	179 (56.1)	0 (0.0)	**< 0.001**

Pathogen distribution calculated among patients with positive cultures (*n* = 147). Percentages for individual pathogens are expressed as proportions of culture-positive patients. ᵃMRSA isolation does not necessarily indicate serious or invasive MRSA infection as defined by TDM indication criterion I1; pathogen-specific TDM eligibility was assessed separately using clinical criteria

No positive cultures were observed in the non-TDM group (*n* = 12), and therefore pathogen distribution is presented only for the overall cohort. Of 179 patients with any concomitant nephrotoxic agent, 174 met the I3 criterion as operationally defined (aminoglycosides, amphotericin B, or intravenous contrast agents); the remaining 5 patients used agents outside this definition (e.g., furosemide, piperacillin-tazobactam) and were therefore not classified under I3

*IQR* Interquartile range, *MRSA* Methicillin-resistant Staphylococcus aureus, *MRCNS* Methicillin-resistant coagulase-negative Staphylococcus, *TDM* Therapeutic drug monitoring

Empirical treatment: vancomycin initiated without a culture result guiding pathogen selection

Targeted treatment: vancomycin continued or initiated based on culture and susceptibility results

Categorical variables compared using Chi-square or Fisher’s exact test

Bold *p* values indicate statistical significance (*p* < 0.05)

**Fig. 2:**
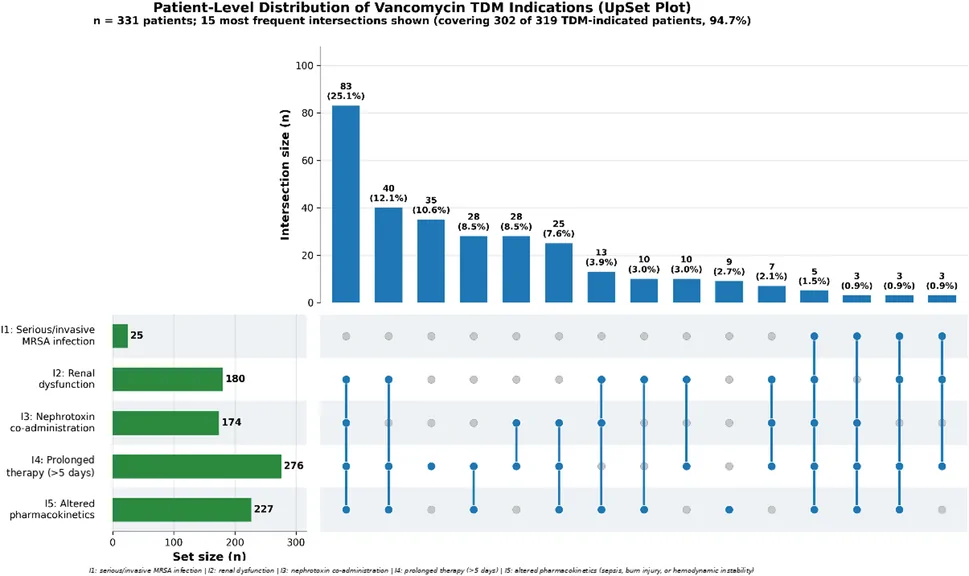
Patient-level distribution of vancomycin therapeutic drug monitoring indications (UpSet Plot). Patient-level distribution of vancomycin therapeutic drug monitoring (TDM) indications among all included patients (*n* = 331). Each column represents a unique combination of co-occurring indications. Upper bars show intersection size (n, %); left horizontal bars show individual set size (n). Filled circles connected by vertical lines indicate active indication combinations; open circles indicate absent indications. The 15 most frequent intersections are displayed, covering 302 of 319 TDM-indicated patients (94.7%). A total of 26 unique indication combinations were identified in the full dataset. I1: Serious/invasive MRSA infection; I2: Renal dysfunction; I3: Nephrotoxin co-administration; I4: Prolonged therapy (observed duration > 5 days during treatment course); I5: Altered pharmacokinetics (sepsis, burn injury, or hemodynamic instability)

Among the included patients, 319 (96.4%) met at least one TDM indication criterion (I1-I5) and were therefore classified as eligible for TDM, whereas 12 patients (3.6%) had no indication. The most common individual TDM indications were prolonged therapy (I4, 83.4%), altered pharmacokinetics (I5, 68.6%), renal dysfunction (I2, 54.4%), nephrotoxic co-administration (I3, 52.6%), and serious or invasive MRSA infection (I1, 7.6%). The median number of TDM indications per patient was 3 (IQR: 2–4). No indications were identified in 12 patients (3.6%), while 49 patients (14.8%) had one indication, 79 (23.9%) had two, 94 (28.4%) had three, 92 (27.8%) had four, and 5 (1.5%) had all five indications (Fig. 2). Fig. 2 summarizes the patient-level overlap of TDM indication criteria. The 15 most frequent combinations covered 302 of 319 TDM-indicated patients (94.7%), and 26 unique indication combinations were identified overall.

When comparing patients according to TDM indication status, several significant differences were observed. Patients meeting TDM indication criteria had a longer ICU length of stay (median 14.0 vs. 7.0 days, *p* = 0.007), higher rates of MV (83.1% vs. 41.7%, *p* = 0.001), and longer duration of MV (11.0 vs. 0.5 days, *p* = 0.003). In addition, sepsis/septic shock (77.4% vs. 25.0%, *p* < 0.001) and central venous catheter use (81.5% vs. 25.0%, *p* < 0.001) were significantly more common among patients meeting TDM indication criteria. Empirical vancomycin use was higher in patients without TDM indication criteria (100.0% vs. 58.0%; *p* = 0.002), while vancomycin treatment duration was significantly longer among those meeting TDM indication criteria (6.0 vs. 2.0 days, *p* < 0.001). No significant differences were observed in age, sex, admission type, or mortality between groups.

Because of the extreme group imbalance (*n* = 319 vs. *n* = 12) and near-universal TDM eligibility, formal multivariable regression modelling was not performed due to limited statistical power and interpretive value.

## Discussion

This study provides a real-world assessment of guideline-informed vancomycin TDM eligibility in an adult ICU setting where vancomycin TDM is not performed. The principal finding is that 96.4% of patients receiving vancomycin met at least one TDM indication criterion derived from the 2020 IDSA/ASHP/PIDS/SIDP consensus recommendations and operationalized for retrospective ICU assessment. This high prevalence indicates that, under these operational criteria, TDM eligibility encompasses the vast majority of ICU patients treated with vancomycin. These findings have important clinical and operational implications for resource planning in settings without structured TDM services.

### Prevalence and profile of TDM eligibility

The proportion of patients meeting TDM eligibility criteria in this study was considerably high. No prior study appears to have systematically quantified the burden of guideline-informed vancomycin TDM eligibility using the 2020 IDSA/ASHP/PIDS/SIDP criteria in a real-world ICU setting where TDM is not performed. Although a high eligibility rate may be expected in critically ill patients, empirical quantification remains important for institutions where TDM infrastructure has not yet been established. Importantly, the present analysis extends beyond a binary eligible/not eligible estimate by mapping the criterion-level composition of TDM eligibility. This provides implementation-relevant evidence on whether monitoring demand is driven mainly by prolonged therapy, altered pharmacokinetics, renal dysfunction, nephrotoxic co-administration, or serious MRSA infection. Accordingly, the main contribution of this study is its role as a needs-assessment and resource-planning analysis in a Turkish tertiary ICU setting where vancomycin TDM is not available, with potential relevance for institutions facing similar reimbursement, laboratory, or infrastructure constraints. Previous surveys have consistently shown that vancomycin TDM is underutilized in clinical practice across diverse healthcare settings. Williams et al. (2023) reported that TDM is applied in only a subset of ICU patients despite its widespread recommendation, while Liebchen et al. (2020) demonstrated substantial variability in TDM implementation across ICUs [19, 20]. This discrepancy highlights a significant gap between monitoring recommendations and real-world practice.

Analysis of individual TDM indications revealed that prolonged therapy (I4) and altered pharmacokinetics (I5) were the most frequent drivers of TDM eligibility. These findings reflect the clinical reality of ICU patients, who commonly receive extended courses of vancomycin and exhibit significant pharmacokinetic variability due to critical illness. Renal dysfunction (I2) and nephrotoxic co-administration (I3) were also highly prevalent, further supporting the need for individualized dosing and monitoring strategies. In contrast, serious or invasive MRSA infection (I1) accounted for only a small proportion of TDM indications, indicating that TDM eligibility in ICU patients is predominantly driven by patient-related clinical factors rather than pathogen-specific considerations. This suggests that restricting TDM considerations to patients with confirmed MRSA infections may overlook many critically ill patients with pharmacokinetic or renal risk factors.

An important methodological consideration is the potential ceiling effect associated with applying guideline-informed TDM criteria in ICU populations. Certain indications, particularly prolonged therapy (I4) and altered pharmacokinetics (I5), are inherently common in critically ill patients and may therefore capture a large proportion of this population. The eligibility criteria used in this study should not be interpreted as a new clinical scoring system, but as guideline-derived criteria operationalized for retrospective ICU assessment. Reporting each criterion separately helps clarify which factors drive eligibility and reduces the risk of interpreting the overall 96.4% rate as a single homogeneous indication. Thus, the high TDM eligibility rate partly reflects the broad and overlapping nature of guideline-informed criteria in ICU practice, while still demonstrating that most vancomycin-treated ICU patients fall within categories where individualized exposure monitoring is considered clinically relevant by international recommendations.

### Exploratory clinical correlates of TDM eligibility

These descriptive differences mainly contextualize the Results rather than provide a separate explanatory model. Because the non-TDM group was very small and TDM eligibility was near-universal, the observed differences should be interpreted as markers of greater illness severity and more complex ICU care among TDM-eligible patients. Formal multivariable modelling was therefore not performed.

### Concordance between real-world practice and guideline recommendations

A key finding of this study is the marked discrepancy between guideline-informed TDM indications and real-world clinical practice in an institution where vancomycin TDM is not performed. Although 96.4% of patients met at least one criterion for TDM eligibility, no included patient underwent vancomycin TDM, and there was no routine AUC-guided monitoring workflow or systematic Bayesian dose-adjustment process in the study setting. This gap reflects a broader issue reported in previous studies, where adherence to vancomycin monitoring recommendations remains inconsistent across institutions [21, 22]. The findings of the present study extend this observation by quantifying the magnitude of guideline-informed monitoring eligibility in a real-world ICU population. The near-universal eligibility observed suggests that, in similar settings, a pragmatic broad or universal monitoring pathway may be more operationally feasible than attempting to identify a small distinct subgroup for TDM. These results support consideration of systematic TDM implementation as a resource-planning and antimicrobial-stewardship priority in ICU practice.

### Implications of empirical versus targeted therapy

An important observation in the present study was that all patients without TDM indication criteria (*n* = 12) received empirical vancomycin therapy, and none had positive cultures. These patients had shorter vancomycin treatment durations (median 2.0 vs. 6.0 days) and lower rates of sepsis, MV, and central venous catheter use. This profile is consistent with the expected clinical scenario of short-course empirical vancomycin use in lower-acuity patients where the drug is discontinued early once cultures return negative or clinical improvement occurs. The contrast between these patients and the TDM-indicated group suggests that TDM eligibility is closely linked to the intensity and duration of vancomycin exposure. Cusumano et al. (2020) emphasized that TDM-guided dosing is particularly relevant in patients with prolonged courses and complex pharmacokinetic profiles, a conclusion supported by the present data [4].

Conversely, 59.5% of the entire cohort received vancomycin empirically, highlighting the widespread use of this antibiotic in the absence of microbiological confirmation. Among patients with TDM indications, 58.0% were on empirical therapy, indicating that TDM eligibility is not restricted to patients with documented MRSA infections. This finding is clinically important because it demonstrates that guideline-informed TDM criteria capture a much broader patient population than those with culture-confirmed Gram-positive infections. Charmillon et al. (2016) similarly reported widespread empirical vancomycin use in French ICUs, with limited TDM implementation despite recognized indications [23].

### Burden of TDM eligibility and resource implications

The near-universal TDM eligibility observed in this study has important implications for healthcare resource planning. If a structured TDM program were implemented, it would need to cover the vast majority of patients receiving vancomycin in the ICU. This represents a substantial clinical workload, including drug level monitoring, dose adjustment, and interdisciplinary coordination.

These findings highlight the need for adequate infrastructure, including laboratory capacity, clinical pharmacy support, and decision-support tools, to enable effective implementation of TDM strategies. Previous studies have emphasized that integrating TDM into routine practice requires not only technical resources but also workflow adaptation and clinician engagement [24, 25]. From an implementation perspective, the integration of TDM in resource-limited settings remains challenging. Barriers such as lack of Bayesian software, limited laboratory capacity, and insufficient clinical pharmacy support may hinder adoption. This study should therefore be interpreted as a real-world clinical pharmacy service needs assessment rather than an evaluation of the clinical effectiveness of TDM.

### Mortality considerations

The overall ICU mortality in this cohort was high, reflecting the severity of illness in this population. Although mortality was numerically higher among patients meeting TDM indication criteria, this difference did not reach statistical significance and should be interpreted with caution given the marked imbalance between groups.

This finding is most likely attributable to underlying disease severity and clinical instability rather than TDM indication status itself. Previous studies have suggested that therapeutic drug monitoring may be associated with improved outcomes in critically ill patients, although this relationship remains complex and influenced by multiple confounding factors [26].

The absence of vancomycin TDM may have important clinical implications, particularly in critically ill patients where both underexposure and overexposure to vancomycin are associated with adverse outcomes. Although causality cannot be established in the present study, the lack of monitoring in a population with a high prevalence of TDM indications raises concerns regarding the absence of individualized exposure monitoring.

However, the observation that the vast majority of critically ill patients met TDM criteria in a setting where vancomycin TDM is not performed highlights a substantial burden of guideline-informed monitoring eligibility. From this perspective, the high prevalence of TDM indications emphasizes the potential operational importance of implementing structured TDM strategies in ICU practice. A prospective pre-post implementation study evaluating clinical, pharmacokinetic, and economic outcomes before and after the introduction of a structured AUC-guided TDM service would be an important next step [27–29].

## Limitations and strengths

This study has several limitations that should be acknowledged. First, the retrospective design may have led to incomplete capture of certain clinical variables and limits causal inference. Second, the study was conducted in a single center, which may restrict the generalizability of the findings to other institutions with different patient populations or clinical practices. Importantly, vancomycin TDM was not performed in the study setting. As a result, actual vancomycin exposure parameters, such as AUC values or trough concentrations, could not be assessed, and no patient-level target attainment or TDM-guided dose adjustment data were available. Therefore, TDM eligibility was determined based on guideline-informed operational criteria rather than measured pharmacokinetic data. While this approach aligns with the study objective, it may not fully reflect real-time dosing adequacy or the clinical effectiveness of TDM.

In addition, some clinically relevant variables, including body mass index and loading dose administration, were not consistently available in the medical records. Augmented renal clearance, ECMO, hypoalbuminemia, and obesity were not formally assessed. Given that these factors are known to influence vancomycin pharmacokinetics, the overall need for TDM may have been underestimated or misclassified in some patients. Furthermore, the small number of patients without TDM indication criteria limited the statistical power of comparative analyses and restricted the interpretation of exploratory clinical comparisons. Additionally, retrospective assignment of TDM indications carries a risk of misclassification bias, as some criteria rely on clinical judgment that may not be fully captured in medical records. The observed high prevalence of TDM eligibility may also partly reflect the broad and inherently overlapping nature of guideline-informed criteria in ICU populations, and should not be interpreted as proof that TDM would improve outcomes in this specific cohort. Finally, given the highly severe case-mix of this tertiary ICU, the external validity of these findings to centers with different patient profiles may be limited.

Despite these limitations, this study provides one of the first real-world evaluations of vancomycin TDM eligibility based on guideline-informed operational criteria in an ICU setting where vancomycin TDM is not performed. The inclusion of all eligible patients enhances the representativeness of the cohort, and the comprehensive assessment of TDM indications offers valuable insight into both the guideline-informed monitoring eligibility burden and the operational implications of implementing structured TDM strategies in intensive care practice.

## Conclusion

In conclusion, the findings of this study demonstrate that the vast majority of ICU patients receiving vancomycin met guideline-informed criteria for TDM eligibility, indicating a substantial burden of guideline-informed monitoring eligibility in a setting where TDM is not available. The predominance of prolonged therapy, altered pharmacokinetics, renal dysfunction, and nephrotoxic co-administration as key drivers of TDM eligibility highlights that monitoring considerations should not be limited to pathogen-specific indications, but should also account for patient-related pharmacokinetic variability in critically ill patients. These findings should be interpreted as evidence of TDM eligibility and resource-planning need, not as proof that TDM implementation would improve outcomes in this cohort.

Although higher mortality was observed among patients meeting TDM indication criteria, this is most likely attributable to underlying disease severity rather than TDM indication status itself. The present study did not evaluate actual vancomycin exposure, AUC target attainment, dose adjustment, nephrotoxicity, microbiological response, treatment failure, mortality, or the clinical effectiveness of TDM. Therefore, the findings should be interpreted as evidence of a high burden of TDM eligibility rather than proof that TDM implementation would improve clinical outcomes in this cohort.

Overall, these findings support consideration of structured TDM implementation in ICU practice and provide real-world evidence to inform resource planning in settings where TDM is currently absent. The generalizability of these findings should be interpreted cautiously. The results primarily reflect a Turkish tertiary ICU in which vancomycin TDM was not available during the study period. Nevertheless, the findings may be informative for institutions facing similar reimbursement, laboratory, or infrastructure constraints, particularly when planning structured vancomycin TDM services. Future prospective pre-post implementation studies are needed to evaluate the clinical, pharmacokinetic, and economic impact of AUC-guided TDM in such settings, with particular attention to outcomes including acute kidney injury incidence, target attainment rates, and total cost of care.

## Supplementary information

Supplementary Material 1.

## Data Availability

The datasets used and/or analysed during the current study are available from the corresponding author upon reasonable request.

## Abbreviations

CRRT: Continuous Renal Replacement Therapy
ICU: Intensive Care Unit
IQR: Interquartile Range
LOS: Length of Stay
MRSA: Methicillin-Resistant Staphylococcus aureus
MRCNS: Methicillin-Resistant Coagulase-Negative Staphylococci
MV: Mechanical Ventilation
TDM: Therapeutic Drug Monitoring
