# Supplementary material for: Guideline-informed vancomycin therapeutic drug monitoring indications in an adult intensive care unit: a retrospective descriptive study
Source: BMC Anesthesiol. 2026 Jul 6;26:580. doi: 10.1186/s12871-026-04078-2 (PMC13617823; doi:10.1186/s12871-026-04078-2)
Supplement: Supplementary file 1 [file 12871_2026_4078_MOESM1_ESM.docx]

**Supplementary Table 1.** Exploratory Univariate and Multivariate Analyses of Clinical Correlates of Vancomycin TDM Indication Status Using Firth Penalized Logistic Regression

| **Variable** | **Univariate OR** | **Univariate 95% CI** | **Univariate p** | **Multivariate OR** | **Multivariate 95% CI** | **Multivariate p** |
| --- | --- | --- | --- | --- | --- | --- |
| **Categorical Variables** | | | | | | |
| Sex (Male) | 1.05 | 0.33 – 3.36 | 0.931 | — | — | — |
| Admission Type (Surgical) | 0.61 | 0.18 – 2.09 | 0.416 | — | — | — |
| Mechanical Ventilation | **6.64** | 2.14 – 20.63 | **0.001** | 2.13 | 0.50 – 9.06 | 0.306 |
| Sepsis/Septic Shock | **9.40** | 2.71 – 32.58 | **<0.001** | 2.65 | 0.45 – 15.74 | 0.284 |
| Central Venous Catheter | **11.88** | 3.26 – 43.27 | **<0.001** | 4.70 | 0.81 – 27.15 | 0.084 |
| Mortality | 3.07 | 0.98 – 9.59 | 0.052 | 0.47 | 0.11 – 1.97 | 0.301 |
| **Continuous Variables** | | | | | | |
| Age (years) | 1.02 | 0.98 – 1.06 | 0.197 | 1.02 | 0.99 – 1.05 | 0.297 |
| ICU LOS (days) | 1.06 | 1.01 – 1.11 | 0.078 | 0.99 | 0.96 – 1.01 | 0.348 |
| Number of Comorbidities | 1.06 | 0.74 – 1.53 | 0.748 | — | — | — |
| MV Duration (days) | 1.06 | 1.00 – 1.12 | 0.085 | 1.01 | 0.98 – 1.04 | 0.434 |

*CI: Confidence interval; ICU: Intensive care unit; LOS: Length of stay; MV: Mechanical ventilation; OR: Odds ratio; TDM: Therapeutic drug monitoring.*
